# A T Cell Receptor Sequencing-Based Assay Identifies Cross-Reactive Recall CD8^+^ T Cell Clonotypes Against Autologous HIV-1 Epitope Variants

**DOI:** 10.3389/fimmu.2020.00591

**Published:** 2020-04-07

**Authors:** Hok Yee Chan, Jiajia Zhang, Caroline C. Garliss, Abena K. Kwaa, Joel N. Blankson, Kellie N. Smith

**Affiliations:** ^1^Bloomberg-Kimmel Institute for Cancer Immunotherapy, Johns Hopkins School of Medicine, Baltimore, MD, United States; ^2^Sidney Kimmel Comprehensive Cancer Center, Johns Hopkins School of Medicine, Baltimore, MD, United States; ^3^Department of Medicine, Johns Hopkins School of Medicine, Baltimore, MD, United States; ^4^Department of Molecular and Comparative Pathobiology, Johns Hopkins School of Medicine, Baltimore, MD, United States

**Keywords:** HIV, cure, elite suppressors, elite controllers, clonotype, CD8 lymphocytes+

## Abstract

HIV-1 positive elite controllers or suppressors control viral replication without antiretroviral therapy, likely via CTL-mediated elimination of infected cells, and therefore represent a model of an HIV-1 functional cure. Efforts to cure HIV-1 accordingly rely on the existence or generation of antigen-specific cytotoxic T lymphocytes (CTL) to eradicate infected cells upon reversal of latency. Detecting and quantifying these HIV-1-specific CTL responses will be crucial for developing vaccine and T cell-based immunotherapies. A recently developed assay, called MANAFEST, uses T cell receptor (TCR) Vβ sequencing of peptide-stimulated cultures followed by a bioinformatic pipeline to identify neoantigen-specific T cells in cancer patients. This assay is more sensitive than conventional immune assays and therefore has the possibility to identify HIV-1 antigenic targets that have not been previously explored for vaccine or T cell immunotherapeutic strategies. Here we show that a modified version of the MANAFEST assay, called ViraFEST, can identify memory CD8^+^ T cell responses against autologous HIV-1 Gag and Nef epitope variants in an elite suppressor. Nine TCR Vβ clonotypes were identified and 6 of these were cross-reactive for autologous variants or known escape variants. Our findings are a proof of principle that the ViraFEST assay can be used to detect and monitor these responses for downstream use in immunotherapeutic treatment approaches.

## Introduction

Antiretroviral therapy (ART) reduces viral load to undetectable levels in the majority of HIV-1-infected patients. Despite this, a persistent latent viral reservoir in tissues and blood prevents complete viral eradication and results in viral rebound upon ART cessation in the vast majority of patients (1). HIV elite suppressors (ES) are patients who control viral replication without ART (2). These patients may represent a model of a functional cure for HIV-1 since many are infected with replication-competent viruses (3, 4) and are thought to control viral replication through HIV-specific CTL (5–8). Thus it may be possible to control the rebound of viremia following the cessation of ART in patients with progressive disease with immunotherapy. One such strategy is the “shock and kill” approach (9), whereby viral replication is induced from within latent reservoirs and endogenously- or exogenously-generated cytotoxic T lymphocytes (CTL) specific for the patient's own virus (autologous virus) kill infected cells. Several immunotherapeutic approaches have been evaluated to induce CTL killing of infected cells, including dendritic cell-based strategies (10), adoptive transfer of CAR T cells (11), and checkpoint inhibition therapy (12).

Identifying antigenic CTL epitopes and evaluating endogenous memory CTL responses will be crucial for developing these immunotherapies into effective treatments. ELISpot, intracellular cytokine staining, and multimer staining have traditionally been employed when assessing HIV-1-specific T cell responses. However, these assays can underestimate the breadth and magnitude of the response (13) and do not enable identification at the clonotypic level, which will be crucial for engineered T cell-based treatments. The mutation associated neoantigen functional expansion of specific T cells (MANAFEST) assay uses peptide-stimulated T cell cultures coupled with T cell receptor Vβ sequencing and a bioinformatic pipeline to identify neoantigen-specific CD8^+^ T cell clonotypes (13–15). This assay has yet to be utilized to evaluate HIV-1-specific responses. This could be particularly useful given the potential for cross-reactivity of HIV-specific T-cell receptors (16–21).

Our goal was to therefore provide a proof-of-principle demonstrating the utility of the MANAFEST assay in identifying T cell responses at the clonotypic level against closely related autologous HIV-1 epitope variants. Elite suppressors are the ideal model for this analysis, owing to well-documented HIV-specific T cell responses and presumed CTL-mediated viral control in these patients. Here we demonstrate that modified use of the MANAFEST assay, called ViraFEST (viral functional expansion of specific T cells), combined with a novel analytical platform can detect cross-reactive CD8^+^ T cell responses to autologous epitope variants in an HIV-1^+^ elite suppressor (ES). This is the first report using this assay to evaluate the cross-reactive nature of T cells specific for autologous HIV-1 epitope variants. Routine use of this assay to detect and monitor T cell responses to HIV-1 antigens could reveal a broad range of immunogenic antigens not previously identified and can identify HIV-1-specific TCRs that could be exploited in vaccine or T cell-based immunotherapies.

## Methods

### Study Patient

Blood samples from the study subject were obtained in 2018 and 2019 after written informed consent and subsequently handled in accordance with protocols approved by the Johns Hopkins University IRB.

### HIV-1 Sequencing and Epitope Selection

Peptides corresponding to previously described autologous variants found in HLA-B^*^57 restricted Gag and Nef proviral, plasma, and replication competent clones were synthesized (22–25). Replication-competent isolates from 2018 were obtained as previously described (3, 4). Briefly, unfractionated CD4^+^ T cells were isolated by negative selection with Miltenyi beads and replicates of 1 million cells were stimulated with PHA (0.5 ug/ml) and 10 million irradiated allogeneic PBMCs. Four million healthy donor lymphoblasts were added on days 2 and 9 to amplify the virus and positive cultures were identified by p24 ELISA (Perkin Elmer). gag and nef were sequenced from 2 isolates as previously described (3, 4).

### T Cell Culture

A total of 25 peptides representing autologous Gag and Nef epitope variants were synthesized by GenScript with a purity of > 85% and used to stimulate T cells in a modified version of the MANAFEST assay (13–15, 26), called ViraFEST. On day 0, peripheral blood mononuclear cells (PBMC) were isolated fresh from whole blood. T cells were isolated using the EasySep Human T Cell Enrichment Kit (Stemcell Technologies). The T cell and T cell-negative fraction were washed, counted, and resuspended at 2.5 × 10^6^/mL in IMDM supplemented with 50 μg/mL gentamicin (ThermoFisher Scientific). The T cell-negative cells were added to a 96-well plate at 100 μL per well. An equal number of T cells was added to each well. Peptide antigens representing the 25 autologous epitope variants were added to individual wells (10 μg/mL), along with a CMV, EBV, and flu peptide pool (1 μg/mL; Miltenyi), and the Ebola virus AY9 epitope, ATAAATEAY, as a negative control, and a condition without peptide to evaluate non-specific T cell expansion. Each culture condition was evaluated in triplicate, with the exception of the ISPRTLNAW peptide, which was done in duplicate, for a total of 83 T cell cultures. An aliquot of 1M T cells was saved as the uncultured baseline condition. Cells were cultured for a total of 10 days at 37°C in a 5% CO_2_ atmosphere. On day 3, half of the culture media was replaced with fresh culture media supplemented with 100 IU/mL IL2, 50 ng/mL IL7, and 50 ng/mL IL15 (for final concentrations of 50 IU/mL IL2, 25 ng/mL IL7, and 25 ng/mL IL15). On day 7, half of the culture media was replaced again with fresh media supplemented with 200 IU/mL IL2, 50 ng/mL IL7, and 50 ng/mL IL15 (for final concentrations of 100 IU/mL IL2, 25 ng/mL IL7, and 25 ng/mL IL15). On day 10, cultured cells were harvested and washed, and CD8+ T cells were isolated using the EasySep Human CD8^+^ T Cell Enrichment Kit (Stemcell Technologies).

### TCR Sequencing, Bioinformatic Processing, and Assessment of Antigen-Specific Expansions

DNA was extracted from each individual cultured CD8^+^ T cell population, as well as the uncultured baseline CD8^+^ T cells, using the QIAamp DNA micro kit (Qiagen). TCR Vβ CDR3 sequencing was performed using the survey sequencing ImmunoSEQ platform (Adaptive Biotechnologies). Conditions with less than 5,000 productive reads after TCR sequencing were excluded from further analysis (Supplementary Table 1). TCR sequencing. tsv files were exported from the ImmunoSEQ analyzer and were uploaded to our publicly-available MANAFEST analysis web app (http://www.stat-apps.onc.jhmi.edu/FEST/). This package prepares TCR sequencing files for analysis, which includes alignment and trimming of nucleotide sequences to obtain only the CDR3 region and removal of nonproductive CDR3 sequences with premature stops or frame-shifts, sequences with an amino acid length <5, and sequences not starting with “C” or ending with “F/W”.

We used a modified version of our statistical criteria to identify antigen-specific T cell clonotypes. To be classified as antigen-specific, a clonotype at the nucleotide level must (1) significantly expand in at least 2 out of 3 replicates relative to the no peptide control well at an FDR of 0.01, (2) only significantly expand in other wells with peptides from the same epitope family using an FDR of 0.01, and (3) have a frequency at least 5 times higher than the frequency of the clone in wells stimulated with peptides from a different epitope family. For visualization, the mean frequency +/- standard deviation of positive and negative expansions was graphed for each well.

## Results

### Clinical Characteristics and Viral Evolution

ES8 is a previously described elite controller who had undetectable viral loads until 5 years prior to this study when he developed persistent low level viremia. The patient's HLA haplotype is A 02/03, B-57/44.

Autologous HIV-1 *gag* and *nef* was sequenced from provirus and plasma obtained in 2004, 2005, 2007, and 2010 and from replication-competent virus cultured in 2006 and 2018 (3, 21–24) as outlined in Table 1. The patient initially had wild type sequence in the HLA-B^*^57 restricted Gag epitopes TW10 and IW9 in proviral clones and in replication-competent virus (3, 22), but he consistently had variants in both epitopes in plasma clones starting in 2004, the earliest time point studied (22). These plasma variants evolved over time (24) and by 2018, replication-competent virus also contained multiple substitutions in both epitopes. A similar discrepancy between proviral and plasma variants was seen in the non-HLA-B^*^57 restricted Gag epitope KK15 and in the HLA-B^*^57 restricted Nef epitope KF9 (22, 23). In contrast there was concordance in sequence between proviral and plasma clones in the HLA-B^*^57 restricted Nef epitopes HW9 and YT9 (23).

**Table 1:** Characteristics of the autologous and known epitopes evaluated for CD8^+^ T cell recognition^*^.

**Protein**	**AA sequence**	**Description**	**References**
GAG TW10 (240-249)	TSTLQEQIGW	Consensus epitope	
		ES8 provirus, 2005, 2007, 2010	(3, 22, 24, 27)
		ES8 replication-competent virus 2006	(28)
	TSTLTEQVAW	ES8 plasma variant 2004 and 2008	(22, 24)
	TSTLAEQVAW	ES8 plasma variant 2008	(24)
	TSTLVEQIAW	ES8 plasma variant 2008	(24)
	TSTLAEQIAW	ES8 plasma variant 2009	(24)
	TSTLSEQVAW	ES8 plasma variant 2009	(24)
	TSTLSEQIAW	ES8 plasma variant 2009	(24)
	TSTLTEQIAW	ES8 plasma variant 2009	(24)
	TSTLQEQIEW	ES plasma variant 2004	(22)
	TSTLAEQMAW	ES8 replication-competent virus 2018	
	TSNLQEQIGW	Common escape mutant	(29–31)
	TSNLQEQIAW	Common escape mutant	(30)
GAG IW9 (147-155)	ISPRTLNAW	Consensus sequence: ES8 provirus 2005	(22, 24)
		ES8 replication-competent virus 2006	
	MSPRTLNAW	ES8 plasma variant, 2004, 2009. RC virus 2018	(22, 24)
GAG KK15 (17-31)	KIRLRPGGKKKYKLK	Consensus sequence. ES8 provirus 2005	(22, 24)
	KIRLRPGGKKRYKLK	ES8 plasma variant 2004.	(22, 24)
		ES8 replication-competent virus 2006	
NEF KF9 (82-90)	KAAVDLSHF	Consensus sequence	
	KSALDLSHF	ES8 provirus variant 2005	(3, 23, 25)
	TAALDMSHF	ES8 plasma variant 2004 and 2010. RC virus 2018	(23, 25)
	KGALDLSHF	Common variant	
	KAALDLSHF	Common variant	
NEF HW9 (116-124)	HTQGYFPDW	Consensus sequence.	
		ES8 replication-competent virus 2018	
	NTQGYFPDW	Previously described escape mutation	(32)
NEF YT9 (120-128)	YFPDWQNYT	Consensus sequence.	
		ES8 replication-competent virus 2018	
	FFPDWQNYT	ES8 provirus and plasma variant (2004, 2010)	(23, 25)

* All peptides except for Gag KK15 represent HLA-B*57 restricted epitopes. Residues highlighted in red are variants of the consensus sequence.

### Evaluation of Memory CD8^+^ T Cell Responses to Autologous Gag and Nef Epitope Variants

**Figure 1:**
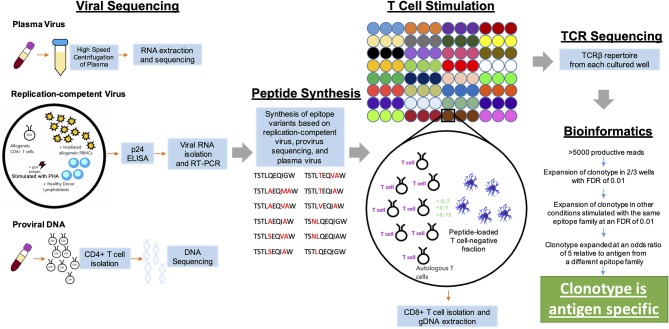
Schematic diagram of the experimental approach. Autologous HIV-1 was sequenced from plasma, replication-competent virus, or proviral DNA. Peptides representing autologous variants of known HLA B57-restricted epitopes were synthesized and used to stimulated T cells in a modified version of the MANAFEST assay. TCR Vβ sequencing was performed on CD8+ T cells from each peptide-stimulated well, as well as uncultured and “no peptide” controls. A bioinformatic pipeline was then used to identify antigen-specific T cell clonotypes.

We next assessed the memory T cell responses against autologous epitope variants using a modified version of the MANAFEST assay, called ViraFEST (Viral Functional Expansion of Specific T cells). A schema of the general experimental approach is shown in Figure 1. Three Gag epitope families were selected for functional validation (Table 1). T cells isolated from PBMC were stimulated with individual peptides. When testing mutation associated neoantigens (MANAs), there is customarily only one epitope tested per mutation (there are rarely 2 nonsynonymous passenger mutations in the same codon). However, owing to the possibility of memory T cells existing against closely-related HIV-1 epitope variants (33, 34), we performed each peptide stimulation in triplicate, with the exception of the ISPRTLNAW peptide, which was evaluated in duplicate, to increase the statistical power of identifying true antigen-specific TCRs for a total of 75 HIV-1 peptide-stimulated cultures. TCR sequencing was performed on DNA extracted from each T cell culture. After TCR sequencing, 5 of the 75 samples were removed from further analysis due to an insufficient number of productive reads (Supplementary Table 1). TCR sequencing files were run through a pre-processing and statistical analysis pipeline to identify antigen-specific T cell clonotypes. The conventional MANAFEST analytical platform relies on several key requirements to confirm antigen specificity, one of which is that the T cell clone must significantly expand in the relevant well relative to every other peptide-stimulated well. Because we evaluated each peptide in triplicate, and considering the potential for T cell cross-reactivity (16, 17), these criteria were modified to evaluate T cell responses to closely-related viral epitope variants where a given T cell clone could be specific for more than one epitope within an epitope family. Specifically, T cell clones should significantly expand in at least 2 out of 3 culture replicates and satisfy all additional criteria as described in the Methods to be considered antigen specific. Given that there are ~1.4 x 10^6^ unique TCR Vβ clonotypes in the memory T cell compartment of a given individual (35), and that we are evaluating responses to 4 different epitope families consisting of 25 unique epitopes, the probability of the same T cell clone expanding in multiple replicates of the same peptide by chance is less than 1.53 × 10^−12^.

**Figure 2:**
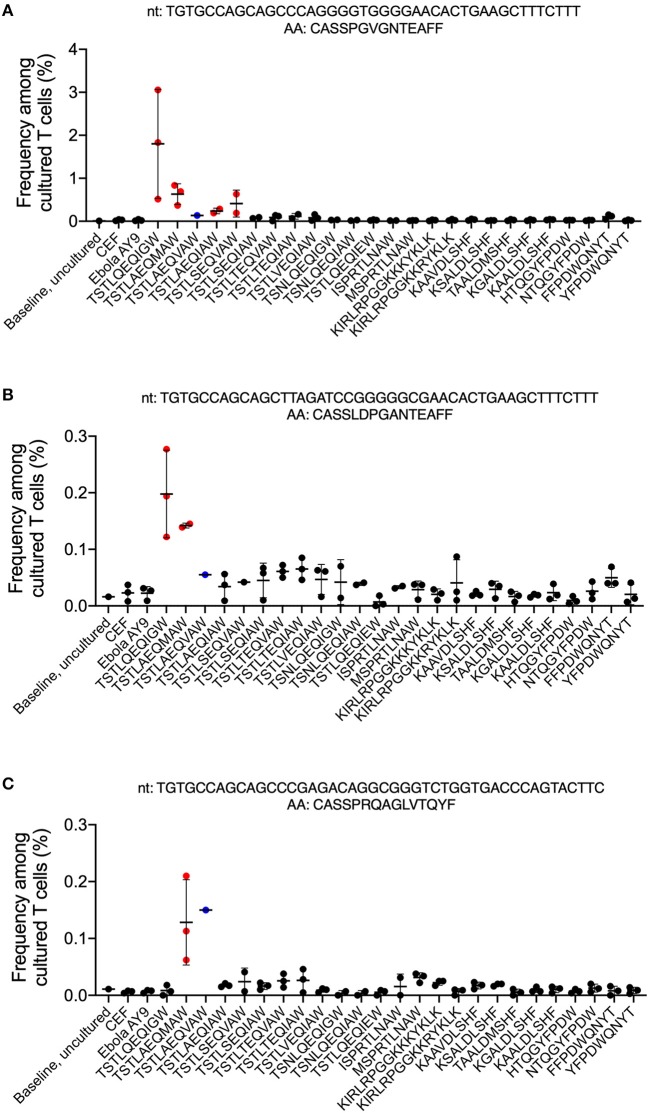
CD8^+^ T cell responses to autologous HIV-1 Gag epitope variants. Peptides representing autologous and known variants of the HIV-1 Gag_240−249_ and Gag_17−31_ HLA-B57-restricted epitopes and the HLA mismatched epitope Gag_17−31_ epitope (Table 1) were synthesized and used to stimulate T cells in the ViraFEST assay. A bioinformatic pipeline that determines antigen specificity identified 3 T cell Vβ clonotypes **(A-C)** that were antigen-specific. The nucleotide (nt) and amino acid (AA) sequence for each clone is shown above their respective graph. The mean frequency (%) after culture +/- standard deviation is shown for each condition where the clonotype contributed to a positive (red) or negative (black) T cell response. In the TSTLAEQIAW-stimulated condition, two of the three triplicates were excluded from analysis, and therefore no determination of a positive or negative response could be determined (blue).

Three T cell clones specific for the Gag p24_235−243_ TW10 epitope family were identified (Figure 2). The first clone, TGTGCCAGCAGCCCAGGGGTGGGGAACACTGAAGCTTTCTTT (CASSPGVGNTEAFF), significantly expanded at an FDR <0.01 and an odds ratio of at least 5 in all three replicates in the T cell culture of the consensus TW10 epitope, TSTLQEQIGW, and satisfied all additional criteria to be considered antigen-specific. This consensus epitope was the predominant proviral variant seen in 2005, 2007, and 2009 (22, 24) (Figure 2A, Supplementary Data 1). Interestingly, this T cell clone was cross-reactive for the TSTLAEQIAW and TSTLSEQVAW variants that were both detected in plasma in 2009 (24) and the variant epitope TSTLAEQMAW that was present in replication-competent isolates cultured in 2018. There was no cross-reactivity with epitopes from other Gag or Nef families or the CEF- or ebola AY9-stimulated control wells. The second clone, TGTGCCAGCAGCTTAGATCCGGGGGCGAACACTGAAGCTTTCTTT (CASSLDPGANTEAFF), was also specific for the consensus TW10 epitope and was cross-reactive for the variant TSTLAEQMAW (Figure 2B, Supplementary Data 1). Although this clone significantly expanded in all three wells stimulated with the TSTLSEQVAW plasma variant from 2009, it only reached the minimum odds ratio threshold in one of the replicates and therefore cannot be considered specific for this antigen. The third clone, TGTGCCAGCAGCCCGAGACAGGCGGGTCTGGTGACCCAGTACTTC (CASSPRQAGLVTQYF) was specific for TSTLAEQMAW (Figure 2C; Supplementary Data 1) and did not demonstrate any cross-reactivity with other epitope family members.

**Figure 3:**
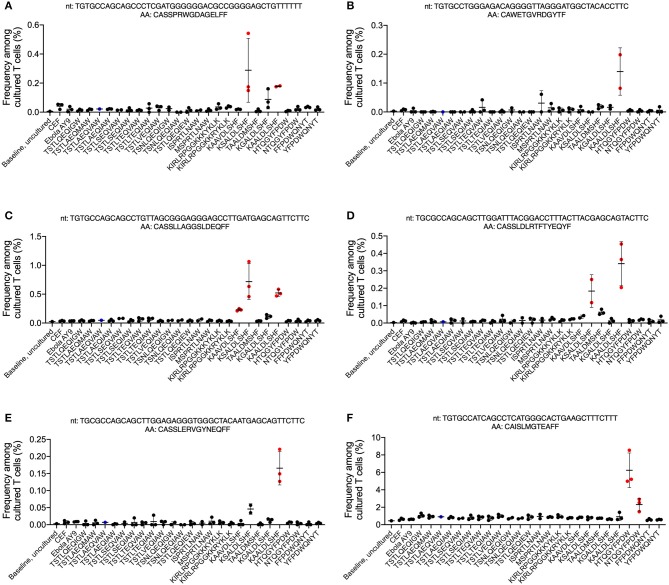
CD8^+^ T cell responses to autologous HIV-1 Nef epitope variants. Peptides representing autologous and known variants of the HIV-1 Nef_82−90_, Nef_116−1204_, and Nef_120−128_ HLA-B57-restricted epitopes (Table 1) were synthesized and used to stimulate T cells in the ViraFEST assay. A bioinformatic pipeline that determines antigen specificity identified 6 T cell Vβ clonotypes **(A–F)** that were antigen-specific. The nucleotide (nt) and amino acid (AA) sequence for each clone is shown above their respective graph. The mean frequency (%) after culture +/- standard deviation is shown for each condition where the clonotype contributed to a positive (red) or negative (black) T cell response. In the TSTLAEQIAW-stimulated condition, two of the three triplicates were excluded from analysis, and therefore no determination of a positive or negative response could be determined (blue).

Five T cell clones specific for the Nef_81−90_ KF9 epitope family and one clone specific for the Nef_116−124_ HW9 epitope family were also identified (Figure 3). The first KF9-specific clone, TGTGCCAGCAGCCCTCGATGGGGGGACGCCGGGGAGCTGTTTTTT (CASSPRWGDAGELFF), recognized KAALDLSHF, a common variant of the consensus epitope and was cross-reactive for the KSALDLSHF variant which was present in proviral clones in 2005 and 2010 (23, 25) (Figure 3A, Supplementary Data 1). The second clone, TGTGCCTGGGAGACAGGGGTTAGGGATGGCTACACCTTC (CAWETGVRDGYTF), only recognized KAALDLSHF (Figure 3B, Supplementary Data 1). The third clone, TGTGCCAGCAGCCTGTTAGCGGGAGGGAGCCTTGATGAGCAGTTCTTC (CASSLLAGGSLDEQFF), demonstrated the most cross-reactivity, recognizing KAAVDLSHF (the consensus epitope), KAALDLSHF, and KSALDLSHF (Figure 3C, Supplementary Data 1). The fourth clone, TGCGCCAGCAGCTTGGATTTACGGACCTTTACTTACGAGCAGTACTTC (CASSLDLRTFTYEQYF), was reactive against KAALDLSHF and KSALDLSHF (Figure 3D, Supplementary Data 1) and the fifth clone, TGCGCCAGCAGCTTGGAGAGGGTGGGCTACAATGAGCAGTTCTTC (CASSLERVGYNEQFF) only recognized KAALDLSHF (Figure 3E, Supplementary Data 1). There were no T cell responses against the TAALDMSHF variant that was present in plasma in 2005 and 2010 (23, 25) and in replication-competent virus from 2018. The only clone recognizing the HW9 family, TGTGCCATCAGCCTCATGGGCACTGAAGCTTTCTTT (CAISLMGTEAFF), was specific for the HTQGYFPDW consensus epitope which was present in replication-competent virus from 2018 and the NTQGYFPDW escape variant (Figure 3F, Supplementary Data 1).

Taken together, these data demonstrate the feasibility of using a modified ViraFEST analytical platform to identify cross-reactive T cell clonotypes in an ES.

## Discussion

This is the first use of the MANAFEST assay to evaluate T cell responses to HIV-1 antigens. This assay, which has been used to identify memory CD8^+^ T cell responses to tumor-derived neoantigens (13–15, 26), uses TCR Vβ sequencing of peptide-stimulated T cell cultures and a bioinformatic pipeline to identify antigen-specific T cell clonotypes. In this proof of concept study, we chose to evaluate HIV-1-specific responses in ES8. ES serve as a model of a T cell-mediated functional cure of HIV. HLA-B^*^57 and HLA-B^*^27 are over-represented in ES (36, 37) and a CTL response that is focused on HLA-B^*^57 restricted epitopes is associated with elite control (36). In prior studies we analyzed HLA-B^*^57 restricted epitopes in proviral and plasma clones from ES and found that while wild-type consensus sequences were present in most proviral clones, plasma clones contained multiple substitutions in these epitopes (22). While some of these substitutions were previously well-described escape mutations such as the T242N mutation in the TW10 Gag 240-249 epitope (29–31), ES8 plasma variants had rare mutations in this epitope that were not recognized by TW10-specific CD8^+^ T cells from other ES. However, CD8^+^ T cells from ES8 and other ES generally recognized autologous plasma variants (22, 28), perhaps explaining why control of viral replication was maintained despite apparent virologic escape. This recognition of autologous TW10 escape variants has previously been reported (22, 27, 38–40), but the mechanism has not been identified. Specifically it's not known whether there is cross-recognition of wild type and escape variants by the same CD8^+^ T cell clonotypes or whether distinct sets of CD8^+^ T cell clonotypes recognize wild type and autologous escape mutants. Differentiating between the two possibilities will be important for developing T cell mediated cure strategies as escape mutants are frequently archived in the latent reservoir (32).

In this study, we identified several T cell clonotypes that recognized several wild type HLA-B^*^57 restricted epitopes as well as autologous variants. Two clones specific for the wild type TW10 epitope cross-reacted with variants that were found in plasma and later in replication-competent virus. A third clone recognized just the most-recent variant that was present in replication-competent virus suggesting that it was a true de novo response to a recent escape variant. In contrast, no detectable responses were made to some prior plasma variants or to epitopes containing the common T242N mutation that was never detected in ES8.

Five clones were specific for a common variant of the Nef KF9 epitope. Although this variant was never detected in any viral clones from ES8, it could have been present in the initial transmitter/founder clone and/or present at a level below the limit of detection. Three of the five CD8^+^ T cell clones cross reacted with the proviral variant of this epitope which may represent an early escape mutation that was archived into the reservoir. Finally, the clone specific for the Nef wild type epitope HW9, cross reacted with NW9 a well characterized escape mutation (41) that was not detected in ES8.

While HIV-1-specific cross-reactivity at the clonotype level has been previously described (18), this is the first use of a TCR immunogenomic platform to identify HIV-1-specific T cell responses. As described previously (13), our MANAFEST-based approach is more sensitive than conventional IFNγ ELISpot assays and has the advantage of being able to identify oligo- and polyclonal T cell responses to antigens. Additionally, once HIV-1-specific clonotypes are identified, the TCR can be used as a molecular barcode to track these antigen-specific cells across biological compartments or in serial blood draws. This provides a benefit for immune monitoring of treatment and therapeutic interventions because the functional assay only needs to be performed once. Our approach is novel and has the potential for broad utility within and outside the HIV research community, however we recognize that this assay is costlier and more bioinformatically-intensive compared to traditional ELISpot assays. Additionally, the limitations of this approach should be considered before being implemented in routine monitoring of immune responses. First, the ViraFEST assay only identifies the Vβ chain corresponding to antigen-specific T cells. Therefore, in order to investigate engineered T cell therapy approaches additional assays would have to be performed to enumerate the Vα chain. Second, the TCR identity does not inform on function or cytokine profile, only that the clonotype is capable of proliferating specifically in response to antigenic stimulation. Although our study serves a proof-of-principle for using this assays when detecting T cell responses against autologous HIV-1 epitope variants, we only studied a single subject and additional studies should be performed to determine if clonotypic cross-reactivity is more broadly seen in ES. Furthermore, while this study was only performed on cells from one time point, the repeatability of this assay has been demonstrated previously in two separate studies using cells from the same patient obtained at different time points for viral antigens (13) and neoantigens (13, 14).

Not only does this serve as the foundation for implementing this assay during routine monitoring of HIV-1-specific responses, but it could be used to identify vaccine or T cell-based immunotherapeutic targets that were not previously identified with conventional immune assays. In this way, determining the clonotypic identity of HIV-1-specific T cells paves the way for engineered T cell therapies that could be used across many patients with common epitope variants and HLA alleles.

## Data Availability Statement

The datasets generated for this study can be found in the Adaptive Biotechnologies ImmuneACCESS Database, https://clients.adaptivebiotech.com/pub/chan-2020-fi.

## Ethics Statement

The studies involving human participants were reviewed and approved by Johns Hopkins IRB. The patients/participants provided their written informed consent to participate in this study. Written informed consent was obtained from the individual(s) for the publication of any potentially identifiable images or data included in this article.

## Author Contributions

HC and CG performed the experiments described here. JZ analyzed the experimental data. AK analyzed the clinical data. JB and KS conceived of the experiments, analyzed the data and wrote the paper.

### Conflict of Interest

KS has received honoraria from Illumina, Inc. and has filed for patent protection for a subset of the technology described herein (serial number 16/341,862). The remaining authors declare that the research was conducted in the absence of any commercial or financial relationships that could be construed as a potential conflict of interest.
